# Altered neuromagnetic activity in default mode network in childhood absence epilepsy

**DOI:** 10.3389/fnins.2023.1133064

**Published:** 2023-03-16

**Authors:** Yingfan Wang, Yihan Li, Fangling Sun, Yue Xu, Fengyuan Xu, Siyi Wang, Xiaoshan Wang

**Affiliations:** Department of Neurology, The Affiliated Brain Hospital of Nanjing Medical University, Nanjing Medical University, Nanjing, China

**Keywords:** spectral power, functional connectivity, interictal, ictal, oscillations, magnetoencephalography, childhood absence epilepsy

## Abstract

**Purpose:**

The electrophysiological characterization of resting state oscillatory functional connectivity within the default mode network (DMN) during interictal periods in childhood absence epilepsy (CAE) remains unclear. Using magnetoencephalographic (MEG) recordings, this study investigated how the connectivity within the DMN was altered in CAE.

**Methods:**

Using a cross-sectional design, we analyzed MEG data from 33 children newly diagnosed with CAE and 26 controls matched for age and sex. The spectral power and functional connectivity of the DMN were estimated using minimum norm estimation combined with the Welch technique and corrected amplitude envelope correlation.

**Results:**

Default mode network showed stronger activation in the delta band during the ictal period, however, the relative spectral power in other bands was significantly lower than that in the interictal period (*p*_*corrected*_ < 0.05 for DMN regions, except bilateral medial frontal cortex, left medial temporal lobe, left posterior cingulate cortex in the theta band, and the bilateral precuneus in the alpha band). It should be noted that the significant power peak in the alpha band was lost compared with the interictal data. Compared with controls, the interictal relative spectral power of DMN regions (except bilateral precuneus) in CAE patients was significantly increased in the delta band (*p*_*corrected*_ < 0.01), whereas the values of all DMN regions in the beta-gamma 2 band were significantly decreased (*p*_*corrected*_ < 0.01). In the higher frequency band (alpha-gamma1), especially in the beta and gamma1 band, the ictal node strength of DMN regions except the left precuneus was significantly higher than that in the interictal periods (*p*_*corrected*_ < 0.01), and the node strength of the right inferior parietal lobe increased most significantly in the beta band (Ictal: 3.8712 vs. Interictal: 0.7503, *p*_*corrected*_ < 0.01). Compared with the controls, the interictal node strength of DMN increased in all frequency bands, especially the right medial frontal cortex in the beta band (Controls: 0.1510 vs. Interictal: 3.527, *p*_*corrected*_ < 0.01). Comparing relative node strength between groups, the right precuneus in CAE children decreased significantly (β: Controls: 0.1009 vs. Interictal: 0.0475; γ 1: Controls:0.1149 vs. Interictal:0.0587, *p*_*corrected*_ < 0.01) such that it was no longer the central hub.

**Conclusion:**

These findings indicated DMN abnormalities in CAE patients, even in interictal periods without interictal epileptic discharges. Abnormal functional connectivity in CAE may reflect abnormal anatomo-functional architectural integration in DMN, as a result of cognitive mental impairment and unconsciousness during absence seizure. Future studies are needed to examine if the altered functional connectivity can be used as a biomarker for treatment responses, cognitive dysfunction, and prognosis in CAE patients.

## 1. Introduction

Early diagnosis is crucial for improving the quality of life of patients with epilepsy, thus, accurate identification of seizure activity has important implications. Although clinicians have historically depended on patients to report the frequency of seizures, patient-reported seizures are known to be unreliable–particularly for young patients (Blum et al., 1996; Tatum et al., 2001). Childhood absence epilepsy (CAE) is an age-dependent idiopathic form of generalized epilepsy. On electroencephalography (EEG), seizures are characterized by a highly recognizable pattern of generalized (bilateral, symmetric, and synchronous) 3 Hz spike and wave discharges (SWDs) (Kessler and McGinnis, 2019). The clinical manifestations of CAE are a sudden disturbance of consciousness, autonomous activity, and language arrest. While collecting clinical data in the past (Wu et al., 2017a), we found that epileptic children struggle to report their experiences and that parents’ records about the frequency, duration, and other important characteristics of seizures are not always reliable, which affects treatment, and efficacy evaluation. Recent studies have shown that 60% of epileptic children experience severe neuropsychiatric disorders, including impairments in attention, cognition, memory, and mood. Even if seizures are pharmacologically controlled, attention deficits may persist for a long time (Crunelli et al., 2020). As children are in a critical stage of growth and development, early identification of CAE is of great significance.

A steady flow of seminal findings advancing our understanding of brain activity continues to emerge from neuroimaging studies (Engel, 2011; Engel et al., 2013; Chen et al., 2021). Studies using functional magnetic resonance imaging (fMRI), which has a high spatial resolution, detect hemodynamic changes in different brain regions in CAE patients by means of blood oxygen level-dependent (BOLD) sequences (Killory et al., 2011; Luo et al., 2011; Xu et al., 2013; Li et al., 2015). However, it is well known that fMRI does not directly measure neural activity, and its signal analysis is confined to a relatively low temporal frequency. Compared to fMRI, electroencephalogram (EEG) and magnetoencephalography (MEG) provide direct measurements of neuronal activity. EEG, which measures the voltage difference in brain electrical activity from the scalp, plays a critical role in both clinical practice and basic research of epilepsy. In recent years, the advent of MEG has made superior spatiotemporal sampling possible without the signal distortion (especially due to the skull) that hampers EEG (Sato et al., 1991). MEG also has excellent spatial resolution of millimeters and temporal resolution of milliseconds, which makes it an attractive modality to assess epileptic activity (Almubarak et al., 2014; Englot et al., 2015; Niso et al., 2015; Hillebrand et al., 2016; Murakami et al., 2016; Nissen et al., 2016). Previous MEG studies on absence epilepsy have typically focused on 3 Hz ictal SWDs. These studies have focused on diagnostic and prognostic biomarkers by measuring source localization and network analysis during ictal periods (Tenney et al., 2013, 2014; Jacobs-Brichford et al., 2014; Miao et al., 2014a,b; Tang et al., 2016; Wu et al., 2017b). Recent studies of the interictal period in CAE patients have reported aberrant interictal brain activity and networks. For example, Chavez et al. (2010) reported that CAE patients have richer connectivity and modularity in the 5–14 Hz range compared to healthy subjects. Xiang et al. (2015) demonstrated that CAE patients have altered interictal brain activity in both low and high-frequency ranges, suggesting that aberrant interictal high-frequency signals are potential new biomarkers. Our early research found that CAE patients display frequency-specific abnormalities in network patterns even during the interictal period (Wu et al., 2017a). Notably, these MEG studies were based on the whole brain. However, unlike other seizure types, absence seizures appear to involve default mode network (DMN) structures before electrographic seizure onset (Bai et al., 2010; Danielson et al., 2011).

The DMN, which comprises the medial frontal cortex (MFC), posterior cingulate cortex (PCC), precuneus (PCu), lateral temporal lobe (LTL), medial temporal lobe (MTL), and inferior parietal lobe (IPL), has been identified as a resting-state functional brain network (Raichle et al., 2001; Cheng et al., 2020). DMN activity is widely accepted to reflect baseline neural activity (Raichle et al., 2001; Havlik, 2017), and the functional connections of specific DMN nodes are considered to be directly related to the level of consciousness. Thus, this extensive endogenous network can serve as a key tool for studying epileptic activity associated with disturbance of consciousness (Vanhaudenhuyse et al., 2010). For these reasons, functional changes in the DMN in children with CAE are worth exploration. Our previous studies demonstrated involvement of the DMN during absence seizures (Miao et al., 2014a), and some DMN nodes in CAE showed frequency-specific abnormalities during interictal periods (Wu et al., 2017a). Therefore, we hypothesized that there are differences in the connectivity patterns of the DMN between CAE patients and controls. We postulated that the resting state is better suited to unravel the functional network difference in CAE since it is not influenced by the strong effects of SWDs and may, thus, be more representative of the underlying pathophysiology. In the present study, to test the hypotheses, EEG, and MEG were simultaneously utilized to acquire data for controls and children with CAE. We then explored the spectral power and functional connectivity of the DMN in CAE.

## 2. Materials and methods

### 2.1. Subjects

**FIGURE 1:**
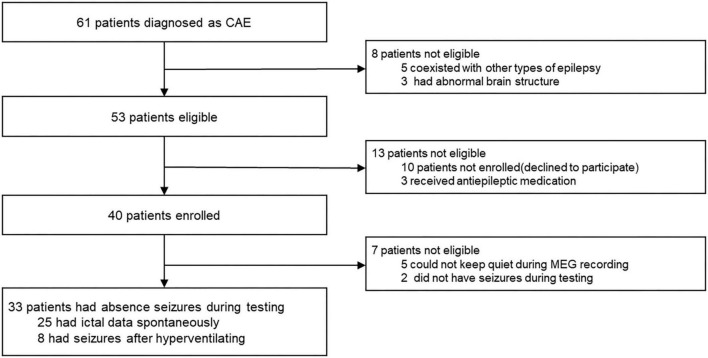
Study profile.

Children (6–12 years old) with newly diagnosed, untreated CAE were recruited from Nanjing Brain Hospital and the Neurology Division at Nanjing Children’s Hospital from October 2020 to January 2022. Although 61 CAE patients were screened, only 33 met the inclusion criteria and were thus included in this study (mean age: 7.7 ± 1.7 years; 20 females and 13 males, the demographic data of the patients are shown in Table 1). In order to clearly show the process of patient inclusion, We added the study profile in Figure 1. An additional 26 healthy children were selected as controls (mean age: 8.3 ± 2.1 years; 16 females and 10 males). The groups were comparable in terms of sex (χ^2^ = 0.005, *p* = 0.942) and age (*F* = 2.404, *p* = 0.282; see demographics in Table 2). This research protocol was approved by the medical ethics committees of Nanjing Medical University, Nanjing Brain Hospital, and Nanjing Children’s Hospital. Informed consent was obtained from all children and their guardians.

**TABLE 1:** Demographic of 33 CAE patients in this study.

Patients	Sex (F/M)	Age (years)	Duration of epilepsy (months)	Seizure frequency (times/day)
1	F	6	1	3–5
2	F	6	3	4–6
3	F	7	2	8
4	F	6	4	6–10
5	F	10	2	5–10
6	F	10	3	10–15
7	M	6	4	3
8	M	10	0.5	2–6
9	F	5	3	4–7
10	M	11	6	3–6
11	F	10	2	5–8
12	F	9	1	5–15
13	M	9	1	3–7
14	F	6	10	5–8
15	F	6	3	2–6
16	M	7.5	6	3–5
17	M	8	3	4–7
18	F	7	7	10
19	M	5	3	6–8
20	F	9	2	5–8
21	F	8	2	3–6
22	M	9	2	3–7
23	F	7	3	5–7
24	M	7	5	3
25	F	8	4	3–5
26	F	8	3	5–8
27	M	10	2	2–6
28	M	9	2	3–8
29	F	6	3	10–15
30	F	10	2	8–10
31	M	6.5	2	5–10
32	F	5	1	5–10
33	M	8	6	10–15

F, female; M, male.

**TABLE 2:** Subjects characteristics and MEG data.

	Total (*n*)	Female, *n* (%)	Age (year), SD	GSWD (> 5 s) during MEG recordings, *n*	Ictal data, *n*	Interictal data, *n*	MEG data from controls, *n*
Patients	33	20 (61)	7.7 (1.7)	65	65	66	–
Controls	26	16 (62)	8.3 (2.1)	–	–	–	52
Comparison*		*p* = 0.942	*p* = 0.282				

*Means *p* < 0.05.

The inclusion criteria were as follows: (1) typical CAE consistent with the International League Against Epilepsy Seizure Classification diagnosed by a neurologist; (2) bilaterally synchronous 3–4 Hz SWDs on a normal background EEG; (3) normal physical and neurological examination; (4) no abnormal brain MRI; and (5) head movement < 5 mm during MEG recordings. The exclusion criteria were: (1) history of other major neurologic or psychiatric diseases or other clinically significant diseases; (2) current intake of antiepileptic medication; and (3) the presence of metal implants. All procedures were performed in accordance with the relevant guidelines and regulations of the Declaration of Helsinki for human experimentation.

### 2.2. MEG recordings

Magnetoencephalographic signals were recorded in a magnetic-shielded room with a whole-head, 275-channel MEG system (VSM Medical Technology Company, Vancouver, BC, Canada) in the MEG Center of Nanjing Brain Hospital. Before data acquisition, three small coils were placed in each person’s left and right pre-auricular and nasion points to measure the participants’ head positions relative to the MEG sensors. All subjects were asked to lie in a supine position comfortably, keep quiet, and stay still with their eyes closed but not to fall asleep (avoid swallowing or teeth clenching). During recording, participants were monitored with an audiovisual system. The system allowed head localization with an accuracy of 1 mm and head movement was limited to 5 mm. For each subject, at least five consecutive epochs with a duration of 2 min were recorded. For CAE patients, if no SWDs were observed in the MEG signals of the first three recorded files, the patients were told to hyperventilate before the fourth MEG recording to provoke seizures. The rate of sampling for data acquisition was 6,000 Hz.

### 2.3. MRI recordings

Three-dimensional structural images were obtained using a 3.0 T MRI scanner (Siemens, Germany). Anatomic 3D T1-weighted images were obtained using a rapid gradient echo sequence (TR/TE = 1,900/2.48 ms). The imaging parameters were as follows: field of view = 250 × 250 mm, flip angle = 9°, and matrix = 512 × 512. For each participant, 176 sagittal slices were collected. All participants were instructed to minimize head movements during the scanning procedure. Markers were placed on the same three fiducial positions used for MEG to co-register the imaging data with the MEG data. All anatomical landmarks digitized in the MEG study were identifiable on MRI.

### 2.4. Data preprocessing

The following strategies were applied to avoid contamination of non-brain or environmental artifacts from spontaneous MEG data: (1) we visually inspected all data for segments containing artifacts caused by head movements or environmental noise and discarded the contaminated segments; (2) notch filters (50 Hz and its harmonics) were used to remove powerline contaminations; and (3) MEG recordings began with a 2 min empty-room recording to capture environment and sensor noise, which was used to calculate the noise covariance for offline source analysis to account for remaining and stationary instrumental, sensor, and environmental noise components. T1-weighted structural volumetric images were automatically reconstructed into the surface model for further source analysis using the FreeSurfer image analysis suite.^1^ We selected 12 DMN-related brain regions as regions of interest (ROI), including the bilateral MFC, PCC, PCu, LTL, MTL, and IPL. Specific Montreal Neurological Institute (MNI) coordinates of the center of each ROI are provided in Supplementary Table 5.

All MEG recordings without prolonged artifacts were retained. For electrographic seizures (generalized 3 Hz SWDs) with a duration of longer than 10 s, the audiovisual system recordings were checked for epileptic seizures to verify ictal periods. The start and end times of seizures were recorded and we chose 5 s in the middle of the seizure as ictal data for further research (because 3–4 Hz SWDs would appear on MEG early or late when patients have clinical manifestations of absence epilepsy, we used the middle data of the ictal period to ensure that the selected data were in the ictal period of absence epilepsy). To choose interictal data without interictal epileptic discharges (IED), we first require “clean” interictal waveforms. The “clean” interictal waveforms were selected in three steps: (1) find a segment of interictal waveform without SWDs; (2) measure the duration of the interictal waveform (>30 s); and (3) check if there were any SWDs before (pre-interval > 30 s) or after (post-interval > 30 s) the interictal waveform. If the duration of the “clean” interictal waveform was longer than 30 s and both pre- and post-intervals were longer than 30 s, we selected 30 s in the middle of the “clean” interictal waveform as the interictal data for further analysis. Each subject in controls was selected two segments of 30 s waveform as control data. The selection process of ictal and interictal data is shown in Supplementary Figure 1.

### 2.5. Minimum norm estimate analysis

We obtained the source-based cortical activation using the depth-weighted minimum-norm estimates (MNE) to estimate the distributed source model of the MEG data, computing the forward solution with a multiple overlapping spheres model, which represents each cortical vertex as a current dipole, including about ∼15,000 vertices. Subsequently, the inverse operator used to estimate the current sources distribution was calculated as follows: (1) the source orientations were constrained to be normal to the cortex surface; (2) the depth weighting algorithm was used to compensate for the inhomogenous sensitivity with depth and orientation of the current flow; and (3) the regularization parameter λ^2^ = 0.33 was used to minimize numerical instability, reduce the sensitivity of MNE to noise, and effectively obtain a spatially smoothed solution, which is defined as the reciprocal of the signal to noise (SNR) of the MEG recordings. The depth-weighted MNE analysis was performed with Brainstorm, a documented program that can be available for free download online under the GNU general public license.^2^

Brainstorm computes a 4 × 4 affine transformation that registers that subject’s T1 MRI to the MNI coordinate system using the *spm_maff8* function from SPM12. Brainstorm sets default coordinates for anatomical fiducials (NAS = nasion, LPA = left ear, RPA = right ear, AC = anterior commissure, PC = Posterior commissure, IH = Inter-hemispheric point) for registration with MEG coordinates. The average current density of all vertices in the ROI was obtained to further estimate the oscillatory power based on the source. We used the Welch method (window duration 5 s with 50% overlap) to calculate the power spectral density (PSD) on each ROI. The PSD values were scaled at each frequency bin relatively to the total power across the entire frequency spectrum: Relative PSD(f) = PSD(f)/∑*_i_* [Total PSD(f*_i_*)], where f*_i_* is the individual frequency from the absolute PSD. This procedure has been shown to standardize PSD values across brain regions and subjects (Niso et al., 2019).

### 2.6. Functional connectivity analysis

Corrected amplitude envelope correlation (AEC-c) analysis was utilized to estimate oscillatory functional connectivity in the aforementioned DMN-related brain regions. Previous studies have reported that AEC-c analysis has strong repeatability and stability in functional connectivity network research (Brookes et al., 2004, 2011). According to the method reported in the previous study, we orthogonalized the signal pairs before envelope computation to eliminate spurious connections due to volume conduction effects and field spread (Colclough et al., 2015). The amplitude envelope was defined as the absolute value of the Hilbert transform of a certain cortical oscillation, obtained from band-pass filtered cortical source activity for each frequency band. This reflects the fluctuation of amplitude over time. AEC-c is calculated by correlating the amplitude envelopes of the cortical oscillatory activities from two ROIs. High AEC-c values indicate synchronous amplitude envelope fluctuations between two ROIs. The AEC-c values for all subjects were computed for all ROIs and the full 12 × 12 adjacency matrix was estimated. The node strength, which was calculated from the sum of the AEC-c values between one ROI and the other 11 ROIs (sum of 11 paired AEC-c values), denotes the functional connectivity within the DMN for each of the 12 ROIs. The relative node strength, i.e., the ratio of the node strength to the total node strength in the current frequency band, represents the proportion of node connectivity in the total connectivity in the current frequency band.

The relative spectral power (i.e., relative PSD) and oscillatory connectivity (i.e., AEC-c) described above categorized by frequency bands: delta (2–4 Hz), theta (5–7 Hz), alpha (8–12 Hz), beta (15–29 Hz), gamma1 (30–59 Hz), and gamma 2 (60–90 Hz).

### 2.7. Statistical analysis

The relative PSD values and AEC-c values were calculated as the measures of spectral power and functional connectivity, respectively. Since the generated relative PSD values and AEC-c values are between 0 and 1, the normality of the data tested by Shapiro–Wilk test didn’t conform to normal distribution, the non-parametric test -Kruskal Wallis test was used in this study to determine its statistical significance. The level of statistical significance was set as *P* < 0.05, corrected for multiple comparisons using the false discovery rate (FDR). All statistical analyses were performed using SPSS 25.0 for Windows (SPSS Inc., Chicago, IL, USA).

## 3. Results

We captured 65 ictal and 66 interictal datasets from the MEG data of 33 children with CAE and 52 control datasets from 26 healthy children.

### 3.1. Relative spectral power analysis

**FIGURE 2:**
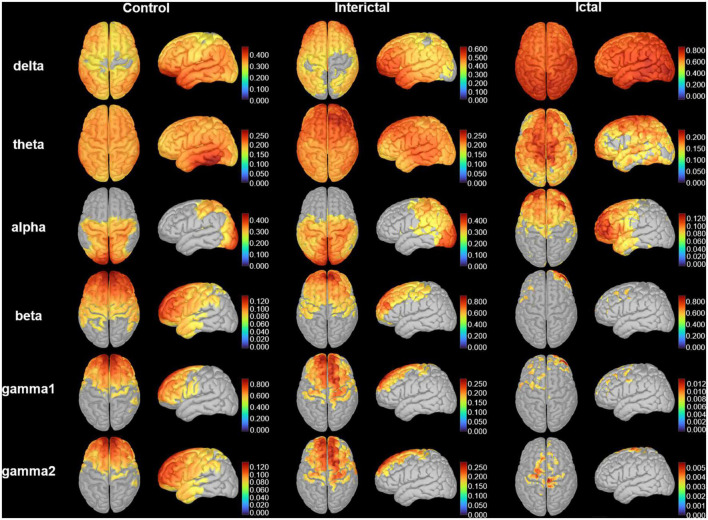
Cortical activation. The distribution of the average cortical activation in control, interictal and ictal data. The activation maps are shown from the top and lateral views. Values range between 0 and 1, indicating the power of cortical signals relatively to the total signal power across the frequency spectrum. Only the cortical sources with a strength larger than 60% of the maximal value are displayed. The current strength for cortical sources is color-coded with large values represented in red.

We calculated the grand-averaged activation on cortical surfaces of bilateral hemispheres in patients and controls (Figure 2). Overall, the activation of the control and interictal data was roughly similar, although activation during the ictal period changed significantly. Ictal cortical activation was mainly characterized by high and extensive activation in the delta band, and significantly decreased activation in the beta, gamma1, and gamma2 bands. Notably, compared with the interictal period, the frontal lobe replaced the parieto-occipital region in cortical activation in the alpha band.

**FIGURE 3:**
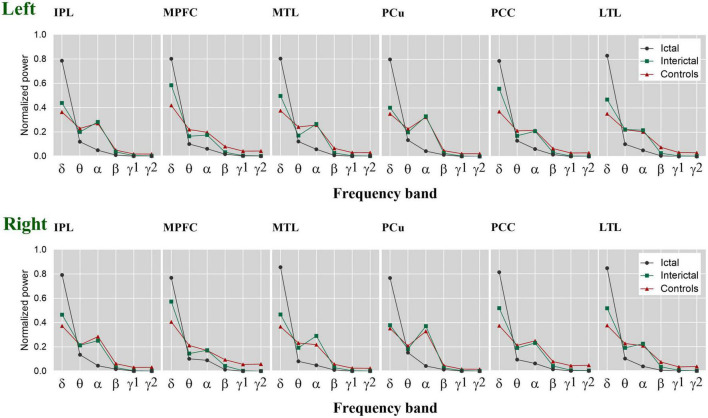
The relative spectral power of each ROI within the DMN. The estimated normalized and averaged spectral power of 12 ROIs in six frequency bands across control, interictal and ictal data, respectively. δ, delta; θ, theta; α, alpha; β, beta; γ 1, gamma 1; γ 2, gamma 2. Left, left hemisphere. Right, right hemisphere.

To clearly show the oscillatory activity patterns (peak power frequency and power distribution), we used line charts to show the DMN relative power of each frequency band in the control, ictal, and interictal data (Figure 3, see Supplementary Tables 1, 2 for the median values and comparisons). Similar to the cortical activation results, the ROIs showed stronger activation in the delta band during the ictal period, however, the relative spectral power in most other bands was significantly lower than that in the interictal period (*p*_*corrected*_ < 0.05 for DMN regions, except bilateral MFC, left MTL, left PCC in the theta band, and the bilateral PCu in the alpha band). It should be noted that the significant power peak in the alpha band was lost compared with the interictal data. However, all ROIs’ relative power of the control and interictal data showed a significant alpha-band power peak, and there is no statistical difference (*p*_*corrected*_ > 0.05). In the delta band, the interictal DMN relative power was higher than that of controls (*p*_*corrected*_ < 0.01 for DMN regions, except bilateral PCu). In the beta-gamma2 band, the situation was the opposite. This result suggests that CAE patients have unique oscillatory DMN activity patterns during the interictal period.

### 3.2. Functional connectivity analysis

**FIGURE 4:**
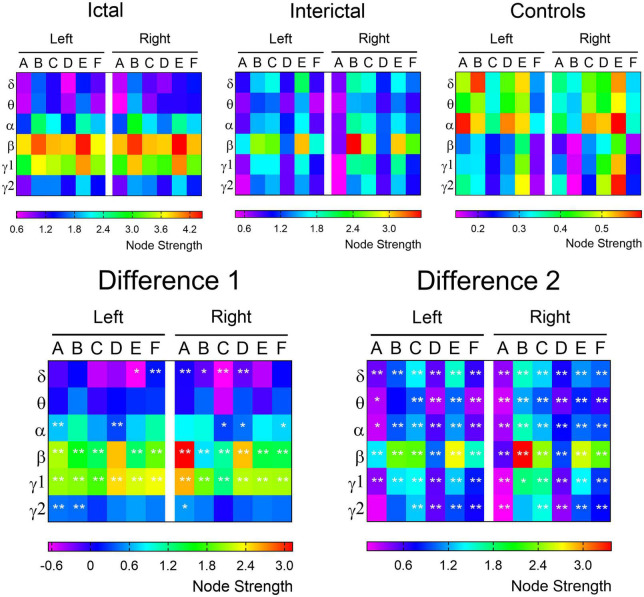
The node strength of each ROI within the DMN. The node strength of each ROI from the delta to gamma 2 bands is color-coded for control, ictal and interictal data, respectively. The difference 1 (ictal minus interictal) and difference 2 (interictal minus control) of the node strength demonstrate the alterations in functional connectivity. A, IPL; B, MFC; C, MTL; D, PCu; E, PCC; F, LTL. *p < 0.05; **p < 0.01.

Next, we calculated the node strength of each ROI in the three data. The difference maps demonstrated the frequency bands and brain regions with significant changes in connectivity within the DMN (Figure 4). In the higher frequency band (alpha-gamma1), especially in the beta and gamma1 band, the ictal node strength of DMN regions except the left PCu was significantly higher than that in the interictal periods (*p*_corrected_ < 0.01), and the node strength of the right IPL increased most significantly in the beta band (Ictal: 3.8712 vs. Interictal: 0.7503, *p*_corrected_ < 0.01). Compared with the controls, the interictal node strength of DMN increased in all frequency bands, especially the node strength of right MFC in beta band (Controls: 0.1510 vs. Interictal: 3.5270, *p*_corrected_ < 0.01).

**FIGURE 5:**
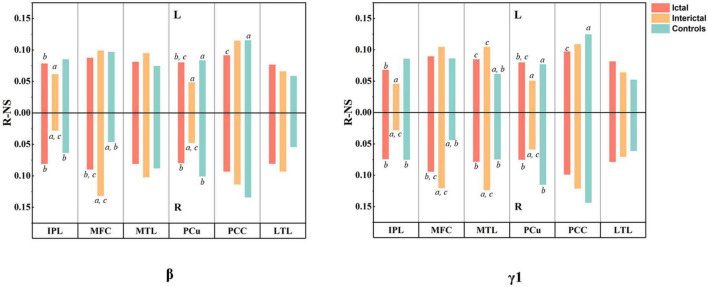
Results of MEG connectivity analysis. The histogram shows the relative node strength of 12 DMN regions in beta and gamma1 bands. The upper half represents the left hemisphere and the lower half represents the right hemisphere. All values are mean. *^a^P* < 0.01 compared to Ictal, *^b^P* < 0.01 compared to Interictal, and *^c^P* < 0.01 compared to controls.

According to the results of difference maps, we focused on the changes in DMN connectivity in the beta and gamma1 bands. The relative node strength of the DMN region was plotted as a histogram (Figure 5, specific values and comparisons are shown in Supplementary Tables 3, 4). Compared with the controls, the interictal data showed a significant increase in the relative node strength of the right MFC and MTL, and a decrease in the bilateral IPL. The PCu and PCC are considered central hubs between DMN node connectivity and consciousness (Vanhaudenhuyse et al., 2010). In the controls, the bilateral PCC and PCu did have high relative node strength, but in the interictal data, the right PCu was no longer the central hub (β: Controls: 0.1009 vs. Interictal: 0.0475; γ 1: Controls: 0.1149 vs. Interictal: 0.0587, *p*_corrected_ < 0.01).

## 4. Discussion

Default mode abnormalities have been reported in various conditions including schizophrenia, depression, Alzheimer’s disease, attention-deficit/hyperactivity disorder, and epilepsy (Broyd et al., 2009). To date, few studies have focused on resting-state neuromagnetic activity in absence epilepsy patients, particularly analysis of interictal duration without IED. This study acquired resting-state MEG data from healthy children and children with CAE during ictal and interictal periods. The relative spectral power and functional connectivity were further analyzed based on region-wise analysis of the DMN.

Ever since Hans Berger described the alpha oscillation in 1929, it has played an important role in varied neuroscientific investigations (Mierau et al., 2017). It has been found that the significant peak of the 8-12 Hz alpha band is a typical brain rhythm in the resting state that mainly occurs in the parieto-occipital lobe and is attenuated by eye opening, visual stimuli, and increased attentiveness. These findings suggest that alpha oscillations seem to be an “idling” rhythm when the brain is alert but still (Adrian and Matthews, 1934). With deepening of such research, this idea has been largely replaced by the inhibition hypothesis, which holds that the amplitude of alpha oscillations reflects the degree of inhibition in the cerebral cortex (Ray and Cole, 1985; Klimesch, 1996). However, increased alpha oscillations have been reported during some internal tasks, such as mental imagery (Cooper et al., 2006), and mental calculation (Palva et al., 2005). Although the specific meaning of the alpha oscillation and underlying mechanism are still unclear, according to the current evidence, the rhythmic activity in the alpha band represents the core characteristics of cortical communication and cognition (Roux et al., 2013). During interictal period without absence seizures, CAE patients had the same posterior-occipital activation as the controls in the alpha band. The distribution of cortical activation is similar to previous studies (Niso et al., 2016, 2019). During absence seizures, the prefrontal lobe replaced the parietal occipital region in the activation of the alpha cortex. Similar to our results, a study of stepwise drug-induced loss of consciousness in healthy volunteers reported that progressive loss of consciousness was closely related to the emergence of a hypersynchronous cortical state in the alpha band (Supp et al., 2011). Notably, this drug induced ongoing alpha activity was widely distributed in the frontal cortex, and modulations of frontal and occipital alpha activity was opposite during loss of consciousness. Besides, previous MEG studies have shown that the frontal cortex to be a critical component in the initiation and propagation of absence seizures (Westmijse et al., 2009; Bai et al., 2010; Tenney et al., 2013). Recent EEG-fMRI studies have shown that the frontal cortex is critical for generating absence seizures (Bai et al., 2010; Moeller et al., 2010; Tenney et al., 2018), and frontal activations could support the cortical focus theory (Meeren et al., 2002). Given these findings, significant activation of the frontal cortex in the alpha band may play a key role in consciousness impairment.

Regarding DMN relative spectral power, we found that the alpha-band power peak played an important role in the maintenance of consciousness, as it existed in the control and interictal data and only disappeared in ictal data with impairment of consciousness. Compared with interictal periods, DMN during ictal periods showed high activation in the delta band, and significantly decreased activation in most other bands. This phenomenon seems to indicate that the disruption of DMN power distribution in various frequency bands is related to the impairment of consciousness during absence seizures. Compared with controls, during interictal periods, children with CAE showed increased DMN relative spectral power in the delta band, accompanied by a decrease in beta-gamma2 band. This result seems to indicate that even in interictal periods without IED, the DMN oscillatory activity patterns in CAE patients are significantly different from those of controls. This finding represents a difference in the power distribution in the whole spectrum. Niso et al. (2016) believe that the spectral power distribution characteristics (i.e., relative PSD) can be used as potential electrophysiological markers. Soriano et al. (2017) used machine learning algorithms to analyze MEG signal features from epileptic patients in interictal period, including total and relative PSD, phase locking value, and phase lag index. They found that the relative PSD of the MEG time-series could distinguish between healthy and epileptic patients with high prediction accuracy. Our findings demonstrated that CAE patients have a unique interictal DMN spectral power distribution compared with controls, which may be a marker for early identification of CAE patients.

In this study, AEC-c analysis was utilized to identify functional connectivity in the DMN. The most striking finding was that interictal DMN connectivity in CAE patients was enhanced compared with controls and further enhanced during ictal periods. Biologically, increased connectivity is a plausible mechanism to explain the generation, or more rapid spread, of spontaneous seizures. According to the difference maps, the most significant difference was in the beta and gamma1 bands–especially the beta band. As a result, our subsequent study was based on these two bands. Beta band was reported previously to have increased connectivity in several MEG and EEG studies in idiopathic/genetic generalized epilepsy, as well as temporal, and frontal lobe epilepsies (Elshahabi et al., 2015). This may be related to the fact that beta is the dominant frequency domain of motor and frontal networks (Papanicolaou et al., 1986). Unlike the results of the MEG studies, in recent years, resting-state fMRI studies have produced consistent results regarding network, and brain regions that are involved in CAE (Luo et al., 2011, 2014). Decreased resting-state functional connectivity within the DMN was found in CAE patients, revealed by both independent compound analysis (ICA) (Li et al., 2015), and the seed-based approach (Yang et al., 2013). Differences in methods lead to differences in results. fMRI detects hemodynamic changes in different brain regions in patients by means of BOLD sequences. MEG measures the magnetic fields induced above the scalp surface by current flow in the brain. However, these results all demonstrated abnormal functional connectivity of DMN in CAE, and these changes may reflect abnormal anatomo-functional architectural integration in DMN, as a result of cognitive impairment, and unconsciousness during absence seizure. Most contemporary neuroscientists consider the PCu and PCC to be the central region of the DMN (Utevsky et al., 2014). We did find that the bilateral PCC and PCu in the control data had high relative node strengths. However, compared with controls, the node strength of the right PCu in CAE patients increased significantly during the ictal and interictal periods, but the relative node strength was low. The PCu is considered a specialized nexus of the DMN that is highly connected to the other DMN regions. Notably, the PCu requires about 35% more glucose than other brain regions, ranking first in terms of metabolic activity among DMN regions (Gusnard et al., 2001). Thus, the decreased relative node strength of the PCu in children with CAE is an abnormal phenomenon that may be related to absence seizures.

This study is subject to several limitations. First, we recruited newly diagnosed CAE patients not yet taking medication. As some parents could not provide accurate seizure frequency and duration information, we could not explore the correlation between these clinical features and brain magnetic signals. In future research, we will aim to educate the parents and improve follow-up to obtain comprehensive and accurate clinical data. Second, we did not explore the relationship between neural network changes and cognitive level in CAE patients. In this study, we aimed to highlight the characteristics of neuromagnetic signals in CAE patients in a specific region. Further research on, cortical activity and clinical characteristics are thus warranted. Lastly, the findings of this study should be considered exploratory. Further longitudinal studies are needed to explore the role of relative PSD and AEC-c in disease progression.

## 5. Conclusion

The results of this study revealed DMN abnormalities in CAE patients, even in interictal periods without interictal epileptic discharges. Specifically, the relative PSD of CAE patients increased in the delta band and decreased in the beta-gamma2 band. In terms of functional connectivity, CAE patients showed enhanced interictal DMN connectivity, especially in the beta and gamma 1 bands, and the relative node strength of the right PCu decreased significantly, and was no longer a central hub. In CAE patients, DMN showed strong activation in delta band during ictal periods, but the relative PSD in other bands was significantly lower than that in interictal periods, with the loss of the alpha power peak. Furthermore, DMN connectivity was significantly higher during absence seizures compared to interictal periods.

Abnormal functional connectivity in CAE may reflect abnormal anatomo-functional architectural integration in DMN, as a result of cognitive mental impairment and unconsciousness during absence seizure. Future studies are needed to examine if the altered functional connectivity can be used as a biomarker for treatment responses, cognitive dysfunction, and prognosis in CAE patients.

## Data availability statement

The raw data supporting the conclusions of this article will be made available by the authors, without undue reservation.

## Ethics statement

The studies involving human participants were reviewed and approved by the Medical Ethics Committee of The Affiliated Brain Hospital of Nanjing Medical University and The Affiliated Children’s Hospital of Nanjing Medical University in China. Written informed consent to participate in this study was provided by the participants’ legal guardian/next of kin.

## Author contributions

YW, YL, and FS designed the study, recruited patients, acquired and analyzed the data, did the literature search, and wrote the report. YX, FX, and SW assisted with the collection of raw data and generation of the figures. XW supervised the administration of the study, collection of data, data analysis, and drafting of the final manuscript. All authors contributed to the article and approved the submitted version.

## Footnotes

1
https://surfer.nmr.mgh.harvard.edu

2
http://neuroimage.usc.edu/brainstorm

## Supplementary material

Supplementary Figure 1 Schematic of ictal and interictal data analysis. MEG waveforms recorded from a CAE patient.
