# Supplementary material for: Altered neuromagnetic activity in default mode network in childhood absence epilepsy
Source: Front Neurosci. 2023 Mar 16;17:1133064. doi: 10.3389/fnins.2023.1133064 (PMC10060817; doi:10.3389/fnins.2023.1133064)
Supplement: Supplementary file 1 [file Table_1.docx]

| Table S1 median values of relative PSD | | | | |  |  |  |
| --- | --- | --- | --- | --- | --- | --- | --- |
| **Brain region** | **group** | relative PSD, median | | | | |  |
|  |  | **δ** | **θ** | **α** | **β** | **γ 1** | **γ 2** |
| Inferior parietal, L | Ictal | 0.7862 | 0.1197 | 0.0498 | 0.0094 | 0.0006 | 0.0001 |
|  | Interictal | 0.4390 | 0.2000 | 0.2821 | 0.0359 | 0.0043 | 0.0027 |
|  | controls | 0.3639 | 0.2288 | 0.2700 | 0.0500 | 0.0184 | 0.0182 |
| Inferior parietal, R | Ictal | 0.7924 | 0.1351 | 0.0458 | 0.0171 | 0.0009 | 0.0001 |
|  | Interictal | 0.4658 | 0.2128 | 0.2510 | 0.0294 | 0.0039 | 0.0023 |
|  | controls | 0.3552 | 0.2088 | 0.2615 | 0.0556 | 0.0238 | 0.0239 |
| Medial frontal, L | Ictal | 0.8017 | 0.1007 | 0.0613 | 0.0172 | 0.0017 | 0.0001 |
|  | Interictal | 0.5845 | 0.1649 | 0.1748 | 0.0333 | 0.0051 | 0.0025 |
|  | controls | 0.4150 | 0.2145 | 0.1862 | 0.0694 | 0.0306 | 0.0313 |
| Medial frontal, R | Ictal | 0.7686 | 0.1020 | 0.0895 | 0.0126 | 0.0008 | 0.0001 |
|  | Interictal | 0.5732 | 0.1455 | 0.1728 | 0.0439 | 0.0036 | 0.0016 |
|  | controls | 0.3945 | 0.2091 | 0.1743 | 0.0926 | 0.0496 | 0.0507 |
| Medial temporal, L | Ictal | 0.8037 | 0.1212 | 0.0570 | 0.0081 | 0.0006 | 0.0001 |
|  | Interictal | 0.4962 | 0.1702 | 0.2655 | 0.0270 | 0.0034 | 0.0020 |
|  | controls | 0.3610 | 0.2298 | 0.2121 | 0.0597 | 0.0232 | 0.0217 |
| Medial temporal, R | Ictal | 0.8565 | 0.0814 | 0.0489 | 0.0105 | 0.0008 | 0.0001 |
|  | Interictal | 0.4672 | 0.1932 | 0.2902 | 0.0294 | 0.0040 | 0.0023 |
|  | controls | 0.3675 | 0.2323 | 0.2188 | 0.0572 | 0.0255 | 0.0248 |
| Precuneus, L | Ictal | 0.7992 | 0.1357 | 0.0446 | 0.0147 | 0.0011 | 0.0001 |
|  | Interictal | 0.4022 | 0.1996 | 0.3317 | 0.0276 | 0.0038 | 0.0016 |
|  | controls | 0.3503 | 0.2231 | 0.3086 | 0.0408 | 0.0158 | 0.0149 |
| Precuneus, R | Ictal | 0.7672 | 0.1527 | 0.0425 | 0.0140 | 0.0010 | 0.0001 |
|  | Interictal | 0.3790 | 0.1825 | 0.3709 | 0.0271 | 0.0043 | 0.0023 |
|  | controls | 0.3518 | 0.2083 | 0.3290 | 0.0475 | 0.0168 | 0.0162 |
| Posterior cingulate, L | Ictal | 0.7869 | 0.1302 | 0.0618 | 0.0165 | 0.0015 | 0.0002 |
|  | Interictal | 0.5578 | 0.1715 | 0.2086 | 0.0341 | 0.0047 | 0.0031 |
|  | controls | 0.3707 | 0.2123 | 0.2158 | 0.0662 | 0.0295 | 0.0302 |
| Posterior cingulate, R | Ictal | 0.8133 | 0.0940 | 0.0617 | 0.0136 | 0.0014 | 0.0002 |
|  | Interictal | 0.5174 | 0.1894 | 0.2305 | 0.0412 | 0.0061 | 0.0037 |
|  | controls | 0.3832 | 0.2102 | 0.2312 | 0.0762 | 0.0403 | 0.0420 |
| Lateral temporal, L | Ictal | 0.8296 | 0.1022 | 0.0525 | 0.0069 | 0.0006 | 0.0001 |
|  | Interictal | 0.4698 | 0.2232 | 0.2167 | 0.0278 | 0.0058 | 0.0033 |
|  | controls | 0.3528 | 0.2197 | 0.2034 | 0.0757 | 0.0324 | 0.0303 |
| Lateral temporal, R | Ictal | 0.8461 | 0.1023 | 0.0379 | 0.0060 | 0.0004 | 0.0000 |
|  | Interictal | 0.5165 | 0.1908 | 0.2245 | 0.0346 | 0.0055 | 0.0025 |
|  | controls | 0.3755 | 0.2259 | 0.2081 | 0.0741 | 0.0339 | 0.0367 |
