# Supplementary material for: Altered neuromagnetic activity in default mode network in childhood absence epilepsy
Source: Front Neurosci. 2023 Mar 16;17:1133064. doi: 10.3389/fnins.2023.1133064 (PMC10060817; doi:10.3389/fnins.2023.1133064)
Supplement: Supplementary file 2 [file Table_2.docx]

| **Brain region** | **Comparison between groups** | Comparison*, corrected *p* | | | | |  |
| --- | --- | --- | --- | --- | --- | --- | --- |
|  |  |  | | | | |  |
|  |  | **δ** | **θ** | **α** | **β** | **γ 1** | **γ 2** |
| Inferior parietal, L | Ictal-Interictal | <0.01 | <0.01 | <0.01 | <0.01 | <0.01 | <0.01 |
|  | Ictal-controls | <0.01 | <0.01 | <0.01 | <0.01 | <0.01 | <0.01 |
|  | Interictal-controls | <0.01 | 0.1562 | 0.6175 | <0.01 | <0.01 | <0.01 |
| Inferior parietal, R | Ictal-Interictal | <0.01 | <0.01 | <0.01 | <0.01 | <0.01 | <0.01 |
|  | Ictal-controls | <0.01 | <0.01 | <0.01 | <0.01 | <0.01 | <0.01 |
|  | Interictal-controls | <0.01 | 1.0000 | 0.8573 | <0.01 | <0.01 | <0.01 |
| Medial frontal, L | Ictal-Interictal | <0.01 | 0.1444 | <0.01 | <0.01 | <0.01 | <0.01 |
|  | Ictal-controls | <0.01 | <0.01 | <0.01 | <0.01 | <0.01 | <0.01 |
|  | Interictal-controls | <0.01 | <0.01 | 0.6048 | <0.01 | <0.01 | <0.01 |
| Medial frontal, R | Ictal-Interictal | <0.01 | 0.6728 | <0.01 | <0.01 | <0.01 | <0.01 |
|  | Ictal-controls | <0.01 | <0.01 | <0.01 | <0.01 | <0.01 | <0.01 |
|  | Interictal-controls | <0.01 | <0.01 | 0.0545 | <0.01 | <0.01 | <0.01 |
| Medial temporal, L | Ictal-Interictal | <0.01 | 0.5923 | <0.01 | <0.01 | <0.01 | <0.01 |
|  | Ictal-controls | <0.01 | <0.01 | <0.01 | <0.01 | <0.01 | <0.01 |
|  | Interictal-controls | <0.01 | <0.01 | 0.6225 | <0.01 | <0.01 | <0.01 |
| Medial temporal, R | Ictal-Interictal | <0.01 | <0.01 | <0.01 | <0.01 | <0.01 | <0.01 |
|  | Ictal-controls | <0.01 | <0.01 | <0.01 | <0.01 | <0.01 | <0.01 |
|  | Interictal-controls | <0.01 | <0.01 | 0.1077 | <0.01 | <0.01 | <0.01 |
| Precuneus, L | Ictal-Interictal | <0.01 | <0.05 | <0.01 | 0.2301 | <0.01 | <0.01 |
|  | Ictal-controls | <0.01 | <0.01 | <0.01 | <0.01 | <0.01 | <0.01 |
|  | Interictal-controls | 0.0774 | <0.01 | 0.2367 | <0.01 | <0.01 | <0.01 |
| Precuneus, R | Ictal-Interictal | <0.01 | <0.01 | <0.01 | 0.1812 | <0.01 | <0.01 |
|  | Ictal-controls | <0.01 | <0.01 | <0.01 | <0.01 | <0.01 | <0.01 |
|  | Interictal-controls | 0.3697 | <0.05 | 0.3140 | <0.01 | <0.01 | <0.01 |
| Posterior cingulate, L | Ictal-Interictal | <0.01 | 0.1648 | <0.01 | <0.01 | <0.01 | <0.01 |
|  | Ictal-controls | <0.01 | <0.01 | <0.01 | <0.01 | <0.01 | <0.01 |
|  | Interictal-controls | <0.01 | <0.01 | 1.0000 | <0.01 | <0.01 | <0.01 |
| Posterior cingulate, R | Ictal-Interictal | <0.01 | <0.01 | <0.01 | <0.01 | <0.01 | <0.01 |
|  | Ictal-controls | <0.01 | <0.01 | <0.01 | <0.01 | <0.01 | <0.01 |
|  | Interictal-controls | <0.01 | <0.05 | 0.3481 | <0.01 | <0.01 | <0.01 |
| Lateral temporal, L | Ictal-Interictal | <0.01 | <0.01 | <0.01 | <0.01 | <0.01 | <0.01 |
|  | Ictal-controls | <0.01 | <0.01 | <0.01 | <0.01 | <0.01 | <0.01 |
|  | Interictal-controls | <0.01 | 0.9640 | 0.9190 | <0.01 | <0.01 | <0.01 |
| Lateral temporal, R | Ictal-Interictal | <0.01 | <0.01 | <0.01 | <0.01 | <0.01 | <0.01 |
|  | Ictal-controls | <0.01 | <0.01 | <0.01 | <0.01 | <0.01 | <0.01 |
|  | Interictal-controls | <0.01 | <0.05 | 0.9213 | <0.01 | <0.01 | <0.01 |

Table S2 relative PSD comparison
