# Supplementary material for: Altered neuromagnetic activity in default mode network in childhood absence epilepsy
Source: Front Neurosci. 2023 Mar 16;17:1133064. doi: 10.3389/fnins.2023.1133064 (PMC10060817; doi:10.3389/fnins.2023.1133064)
Supplement: Supplementary file 3 [file Table_3.docx]

Table S3 relative node strength values

| **Brain region** | **group** | **β** | | **γ 1** | |  |
| --- | --- | --- | --- | --- | --- | --- |
|  |  |  |  |  |  |  |
|  |  | ns, mean | r-ns | ns, mean | r-ns |  |
| Inferior parietal, L | Ictal | 3.7549 | 0.0784 | 2.7816 | 0.0679 |  |
|  | Interictal | 1.6439 | 0.0615 | 0.7253 | 0.0455 |  |
|  | controls | 0.2774 | 0.0850 | 0.3162 | 0.0857 |  |
| Inferior parietal, R | Ictal | 3.8712 | 0.0808 | 3.0380 | 0.0741 |  |
|  | Interictal | 0.7503 | 0.0281 | 0.4479 | 0.0281 |  |
|  | controls | 0.2066 | 0.0633 | 0.2769 | 0.0751 |  |
| Medial frontal, L | Ictal | 4.1894 | 0.0874 | 3.6678 | 0.0895 |  |
|  | Interictal | 2.6432 | 0.0989 | 1.6694 | 0.1046 |  |
|  | controls | 0.3162 | 0.0968 | 0.3181 | 0.0862 |  |
| Medial frontal, R | Ictal | 4.3003 | 0.0898 | 3.8592 | 0.0942 |  |
|  | Interictal | 3.5270 | 0.1319 | 1.9205 | 0.1204 |  |
|  | controls | 0.1510 | 0.0462 | 0.1610 | 0.0437 |  |
| Medial temporal, L | Ictal | 3.8848 | 0.0811 | 3.4768 | 0.0848 |  |
|  | Interictal | 2.5310 | 0.0947 | 1.6696 | 0.1046 |  |
|  | controls | 0.2435 | 0.0746 | 0.2266 | 0.0614 |  |
| Medial temporal, R | Ictal | 3.8712 | 0.0808 | 3.2132 | 0.0784 |  |
|  | Interictal | 2.7315 | 0.1022 | 1.9686 | 0.1234 |  |
|  | controls | 0.2870 | 0.0879 | 0.2751 | 0.0746 |  |
| Precuneus, L | Ictal | 3.8406 | 0.0802 | 3.2748 | 0.0799 |  |
|  | Interictal | 1.2970 | 0.0485 | 0.8088 | 0.0507 |  |
|  | controls | 0.2721 | 0.0833 | 0.2836 | 0.0769 |  |
| Precuneus, R | Ictal | 3.8181 | 0.0797 | 3.0810 | 0.0752 |  |
|  | Interictal | 1.2690 | 0.0475 | 0.9361 | 0.0587 |  |
|  | controls | 0.3293 | 0.1009 | 0.4237 | 0.1149 |  |
| Posterior cingulate, L | Ictal | 4.3860 | 0.0915 | 3.9869 | 0.0973 |  |
|  | Interictal | 3.0619 | 0.1145 | 1.7373 | 0.1089 |  |
|  | controls | 0.3765 | 0.1153 | 0.4596 | 0.1246 |  |
| Posterior cingulate, R | Ictal | 4.4533 | 0.0930 | 4.0458 | 0.0987 |  |
|  | Interictal | 3.0338 | 0.1135 | 1.9324 | 0.1211 |  |
|  | controls | 0.4379 | 0.1341 | 0.5297 | 0.1436 |  |
| Lateral temporal, L | Ictal | 3.6642 | 0.0765 | 3.3392 | 0.0815 |  |
|  | Interictal | 1.7622 | 0.0659 | 1.0171 | 0.0637 |  |
|  | controls | 0.1910 | 0.0585 | 0.1928 | 0.0523 |  |
| Lateral temporal, R | Ictal | 3.8763 | 0.0809 | 3.2219 | 0.0786 |  |
|  | Interictal | 2.4879 | 0.0930 | 1.1231 | 0.0704 |  |
|  | controls | 0.1767 | 0.0541 | 0.2253 | 0.0611 |  |

^†^ns, node strength; r-ns, relative node strength
