# Supplementary material for: Altered neuromagnetic activity in default mode network in childhood absence epilepsy
Source: Front Neurosci. 2023 Mar 16;17:1133064. doi: 10.3389/fnins.2023.1133064 (PMC10060817; doi:10.3389/fnins.2023.1133064)
Supplement: Supplementary file 4 [file Table_4.docx]

Table S4 relative node strength comparison

| **Brain region** | **Comparison between groups** | Comparison*, corrected *p* | |
| --- | --- | --- | --- |
|  |  | **β** | **γ 1** |
| Inferior parietal, L | Ictal-Interictal | <0.01 | <0.01 |
|  | Ictal-controls | 0.0813 | 0.5256 |
|  | Interictal-controls | 1.0000 | 0.0277 |
| Inferior parietal, R | Ictal-Interictal | <0.01 | <0.01 |
|  | Ictal-controls | 0.0094 | 0.4668 |
|  | Interictal-controls | <0.01 | <0.01 |
| Medial frontal, L | Ictal-Interictal | 1.0000 | 1.0000 |
|  | Ictal-controls | 1.0000 | 1.0000 |
|  | Interictal-controls | 1.0000 | 0.0230 |
| Medial frontal, R | Ictal-Interictal | <0.01 | <0.01 |
|  | Ictal-controls | <0.01 | <0.01 |
|  | Interictal-controls | <0.01 | <0.01 |
| Medial temporal, L | Ictal-Interictal | 0.6885 | 0.0926 |
|  | Ictal-controls | 0.8551 | <0.01 |
|  | Interictal-controls | 1.0000 | <0.01 |
| Medial temporal, R | Ictal-Interictal | 0.2421 | <0.01 |
|  | Ictal-controls | 0.1530 | 0.0150 |
|  | Interictal-controls | 0.1035 | <0.01 |
| Precuneus, L | Ictal-Interictal | <0.01 | <0.01 |
|  | Ictal-controls | <0.01 | <0.01 |
|  | Interictal-controls | 0.5040 | 1.0000 |
| Precuneus, R | Ictal-Interictal | <0.01 | <0.01 |
|  | Ictal-controls | 0.0255 | 1.0000 |
|  | Interictal-controls | <0.01 | <0.01 |
| Posterior cingulate, L | Ictal-Interictal | 0.0678 | 1.0000 |
|  | Ictal-controls | 0.0346 | 1.0000 |
|  | Interictal-controls | 1.0000 | 1.0000 |
| Posterior cingulate, R | Ictal-Interictal | 0.1663 | 0.0533 |
|  | Ictal-controls | 0.5040 | 1.0000 |
|  | Interictal-controls | 0.3650 | <0.01 |
| Lateral temporal, L | Ictal-Interictal | 0.0231 | 0.0342 |
|  | Ictal-controls | <0.01 | <0.01 |
|  | Interictal-controls | 0.0432 | 0.0371 |
| Lateral temporal, R | Ictal-Interictal | 0.0601 | 0.0521 |
|  | Ictal-controls | 0.0263 | 0.0347 |
|  | Interictal-controls | 0.0242 | 0.0700 |
