# Supplementary material for: Altered neuromagnetic activity in default mode network in childhood absence epilepsy
Source: Front Neurosci. 2023 Mar 16;17:1133064. doi: 10.3389/fnins.2023.1133064 (PMC10060817; doi:10.3389/fnins.2023.1133064)
Supplement: Supplementary file 5 [file Table_5.docx]

Table S5 MNI coordinates (x, y, z) of the center of each DMN-related brain region

|  | Left hemisphere (mm) | Right hemisphere (mm) |  |
| --- | --- | --- | --- |
|  |  |  |  |
| Medial frontal cortex | (-3.0, 36.8, -17.9) | (6.8, 32.0, -16.7) |  |
| Posterior cingulate cortex | (-7.0, -43.2, 19.6) | (5.6, -42.2, 17.7) |  |
| Precuneus | (-9.0, -61.5, 41.0) | (9.4, -60.3, 39.3) |  |
| Lateral temporal lobe | (-57.4, -27.7, -12.7) | (56.3, -31.5, -9.9) |  |
| Medial temporal lobe | (-20.5, -32.4, -19.1) | (20.4, -33.0, -17.5) |  |
| Inferior parietal lobe | (-41.1, -64.9, 34.5) | (43.5, -63.1, 34.8) |  |
