# Supplementary figures and images for: Altered neuromagnetic activity in default mode network in childhood absence epilepsy
Source: Front Neurosci. 2023 Mar 16;17:1133064. doi: 10.3389/fnins.2023.1133064 (PMC10060817; doi:10.3389/fnins.2023.1133064)

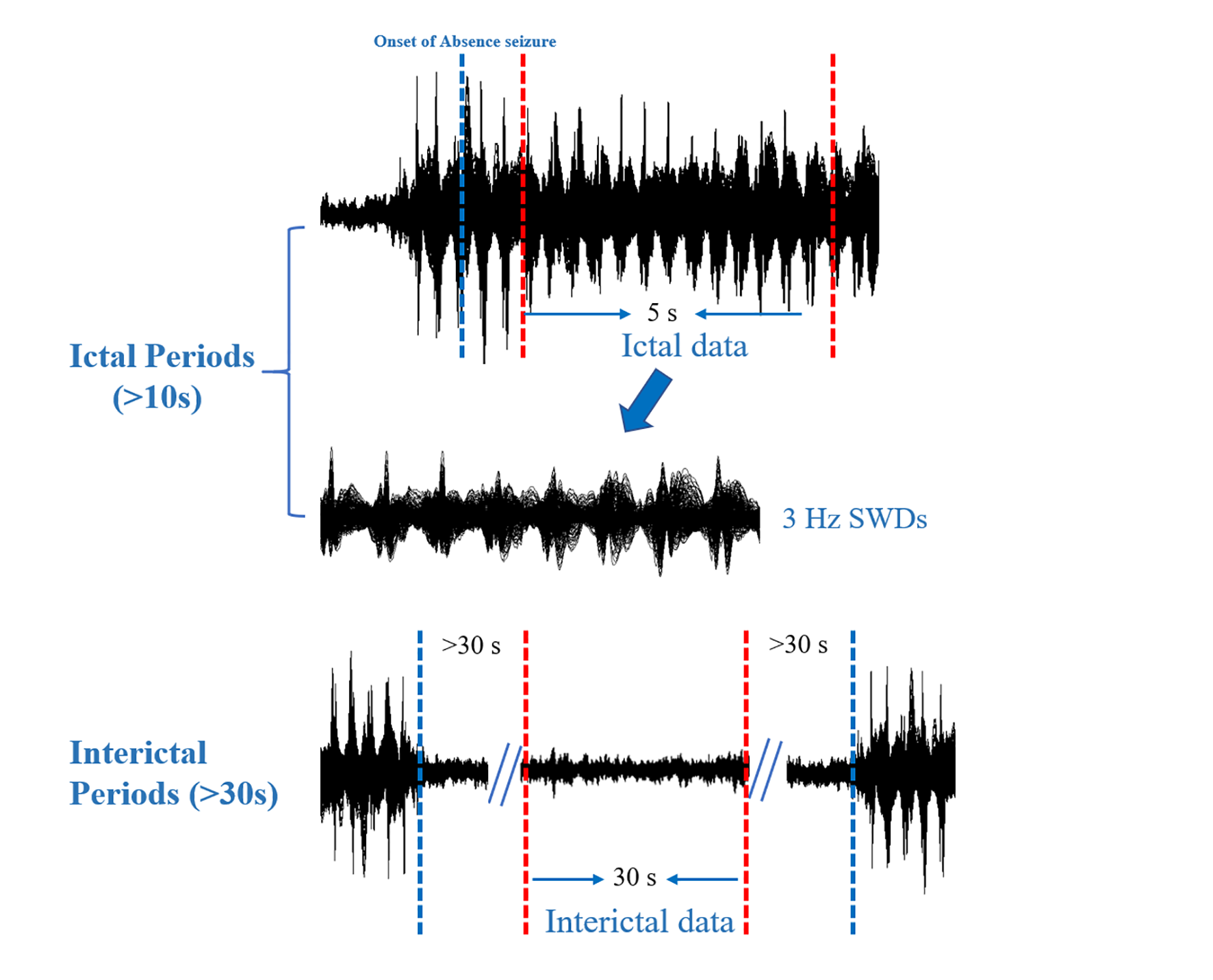

Supplement: Supplementary Figure 1 — Schematic of ictal and interictal data analysis. MEG waveforms recorded from a CAE patient. [file Image_1.TIF]
